# Comments on the Research Society Meeting

**Published:** 1918-09

**Authors:** 


### COMMENTS ON THE RESEARCH SOCIETY MEETING.

At the last meeting of the Research Society of the American Red Cross in France on July 26th and 27th, a number of subjects of great interest and importance to medical officers were discussed. A full report of the meeting is published in this number of *War Medicine*, but, as has been suggested, editorial comments may call attention to and stress important points brought out in the discussions, thus perhaps adding interest to them.

